# Supplementary material for: WNK regulates Wnt signalling and β-Catenin levels by interfering with the interaction between β-Catenin and GID
Source: Commun Biol. 2020 Nov 12;3:666. doi: 10.1038/s42003-020-01386-2 (PMC7665214; doi:10.1038/s42003-020-01386-2)
Supplement: Supplementary file 1 — Supplementary Information [file 42003_2020_1386_MOESM1_ESM.pdf]

Supplementary information

WNK regulates Wnt signalling and β-Catenin levels by interfering with the interaction between β-Catenin and GID

Atsushi Sato, Masahiro Shimizu, Toshiyasu Goto, Hiroyuki Masuno, Hiroyuki Kagechika, Nobuyuki Tanaka and Hiroshi Shibuya

Supplementary Figure 1

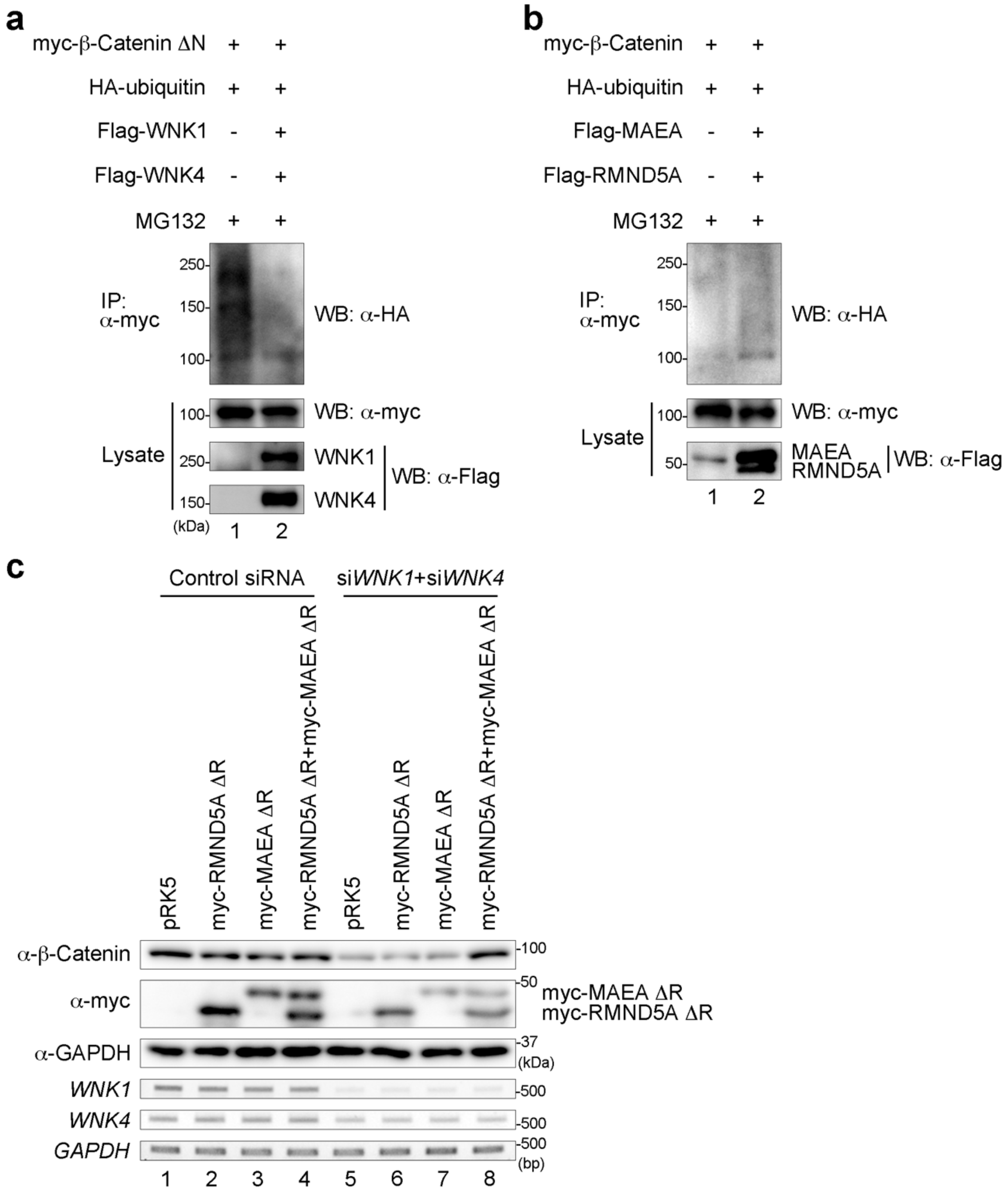

Supplementary Figure 1. WNK regulates the ubiquitination or protein level of β-Catenin through the GID complex. (a) Western blot analysis of ubiquitinated β-Catenin ΔN following the expression of WNK1 and WNK4 in HEK293T cells. (b)

Western blot analysis of ubiquitinated β-Catenin following the expression of RMND5A and MAEA in HEK293T cells. (c) Western blot analysis of endogenous β-Catenin following the knockdown of both *WNK1* and *WNK4*, or the expression of RMND5A ΔR and/or MAEA ΔR in SW480 cells.

Supplementary Figure 2

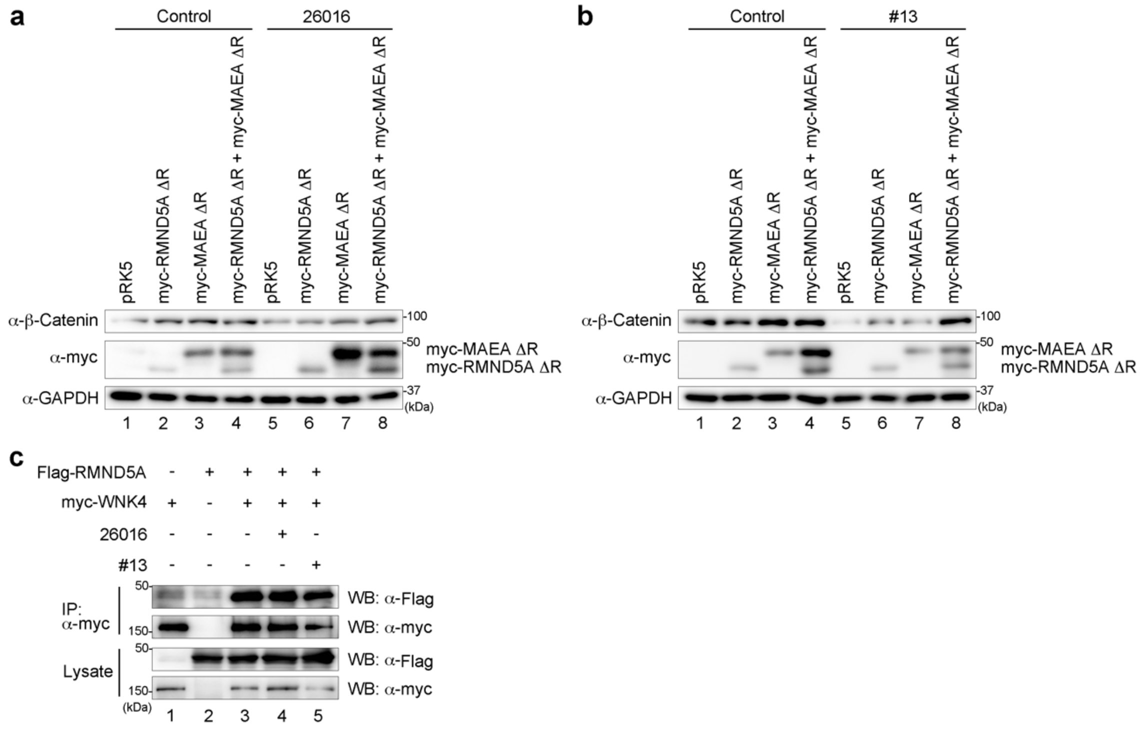

Supplementary Figure 2. The dominant-negative form of the GID complex could rescue the reduction of β-Catenin by WNK inhibitors. (a-b) Western blot analysis of endogenous β-Catenin following treatment with the WNK inhibitor 26016 (a) or #13 (b), or the expression of RMND5A ΔR and/or MAEA ΔR in SW480 cells. (c) The interaction between WNK4 and RMND5A following treatments with WNK inhibitors (26016 and #13) was examined in HEK293T cells by co-immunoprecipitation.

Supplementary Figure 3

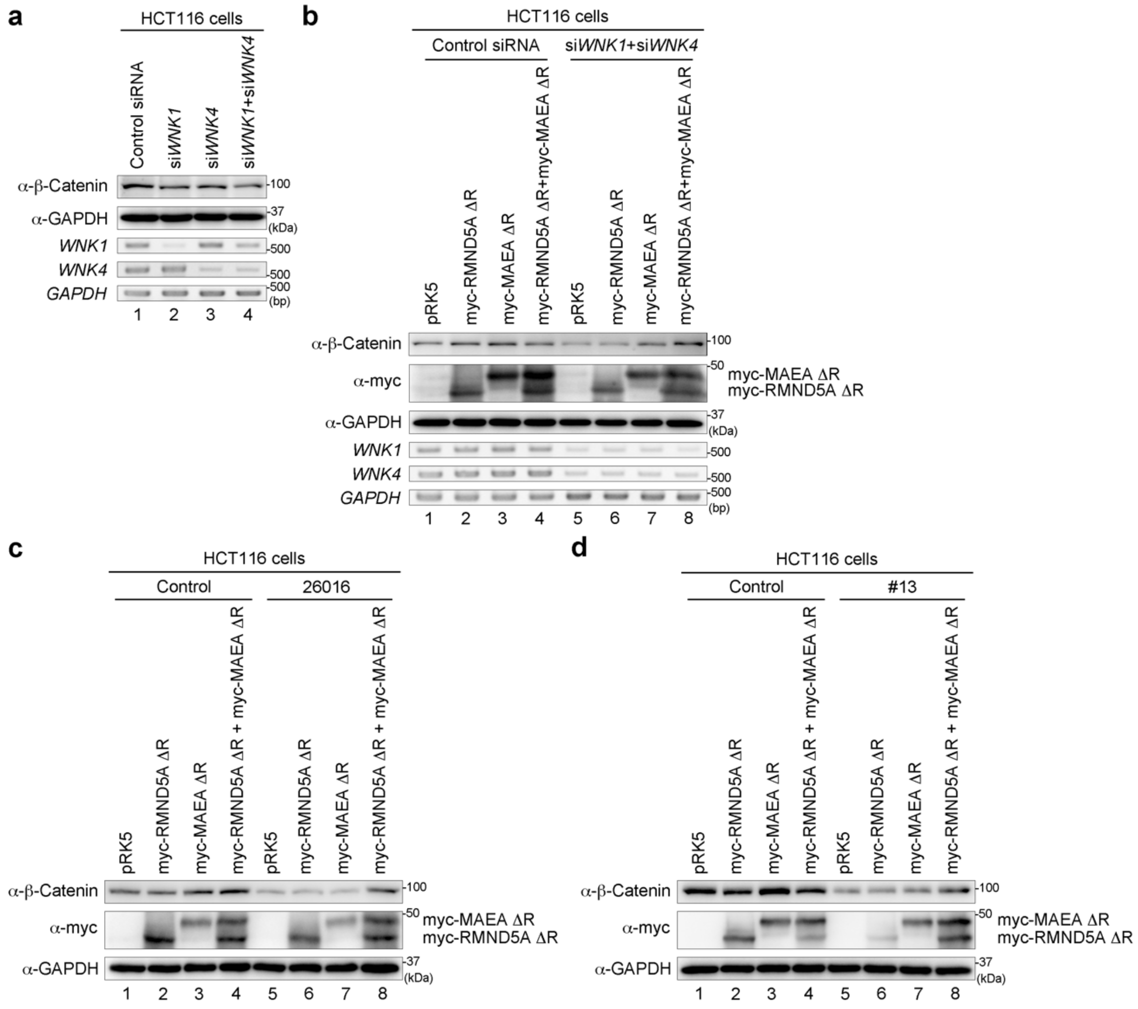

Supplementary Figure 3. WNK regulates the protein level of β-Catenin in HCT116 colorectal cancer cells. (a) Western blot analysis of endogenous β-Catenin following the knockdown of *WNK1* and/or *WNK4* in HCT116 colorectal cancer cells. (b-d) Western blot analysis of endogenous β-Catenin following the knockdown of both *WNK1* and *WNK4* (b), treatment with the WNK inhibitor 26016 (c) or #13 (d), or the expression of RMND5A ΔR and/or MAEA ΔR in HCT116 colorectal cancer cells.

Supplementary Figure 4

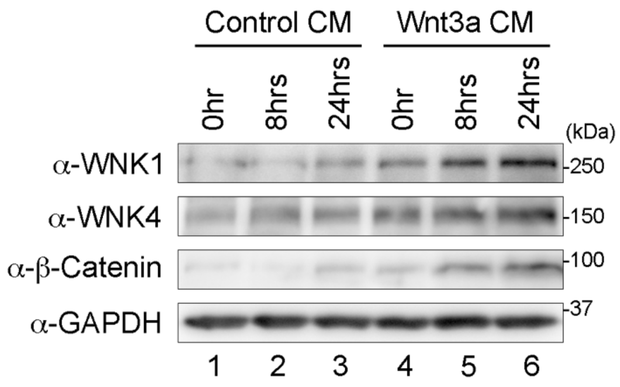

Supplementary Figure 4. Wnt regulates the protein level of both WNK1 and WNK4. Western blot analysis of endogenous protein expression with or without Wnt stimulation in HEK293T cells.

Supplementary Figure 5

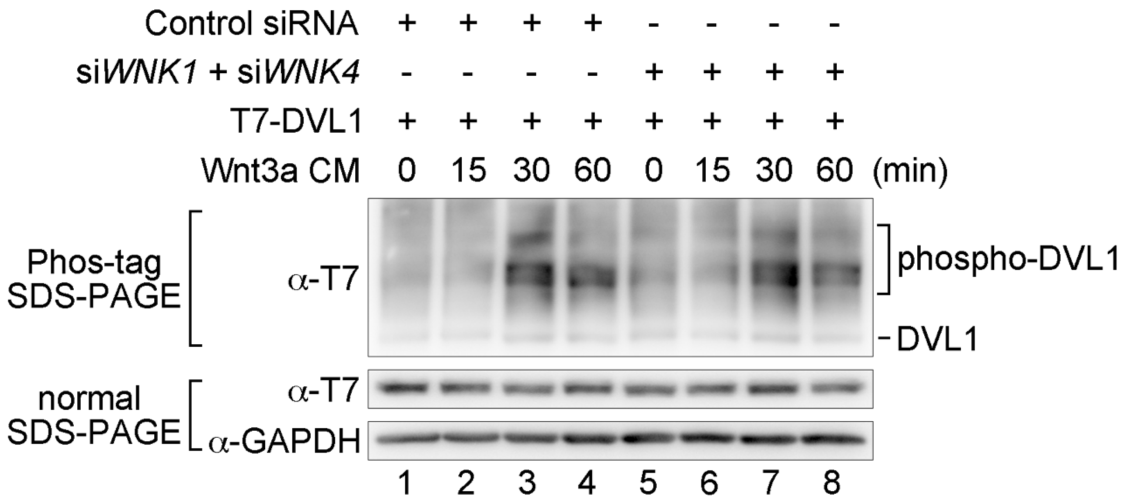

Supplementary Figure 5. WNK did not affect Dvl1 phosphorylation by Wnt stimulation. Western blot analysis of T7-DVL1 phosphorylation using Phos-tag SDS-PAGE following treatments of Wnt stimulation or the knockdown of both *WNK1* and *WNK4* in HEK293T cells.

Supplementary Figure 6

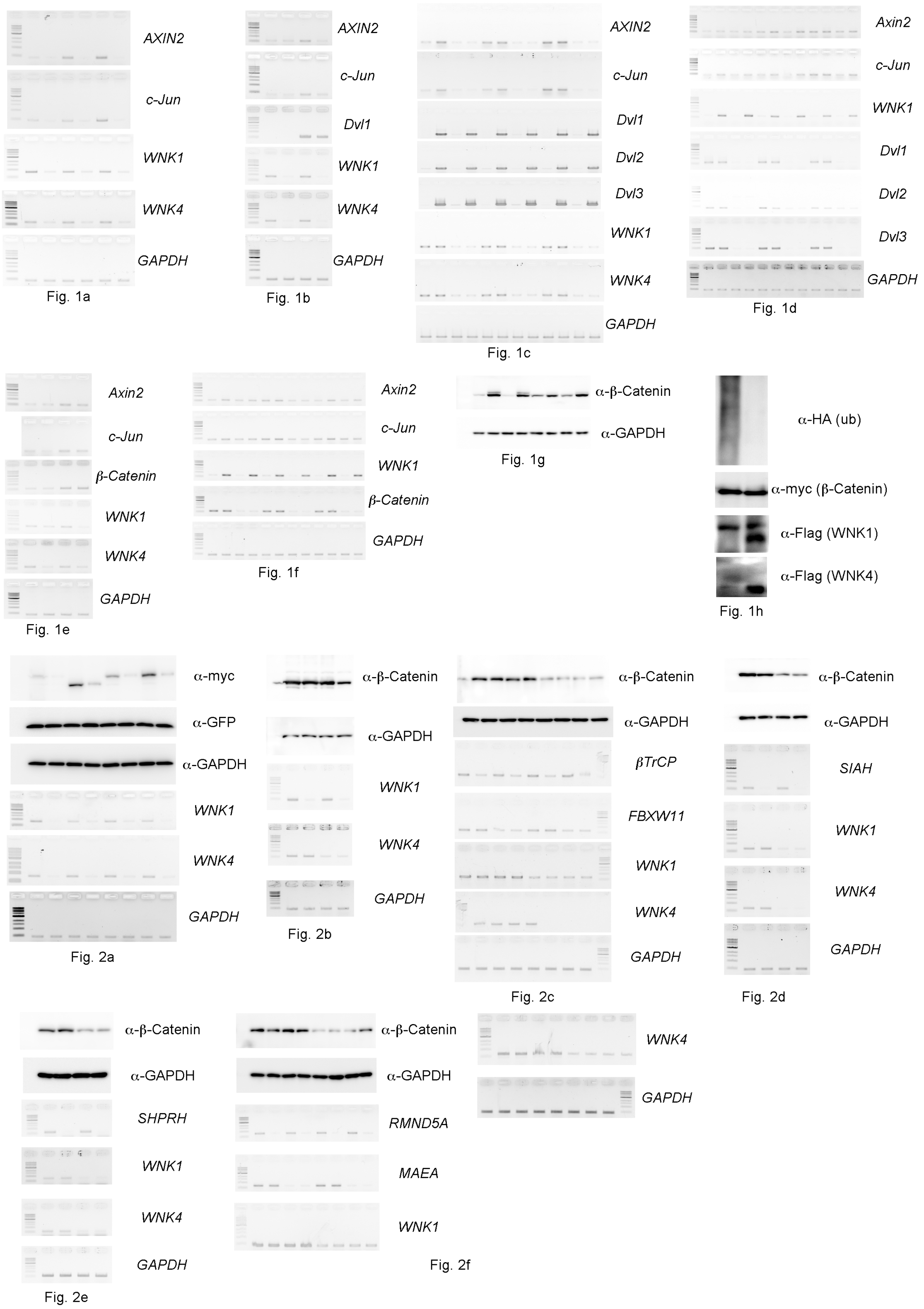

Supplementary Figure 6 continued

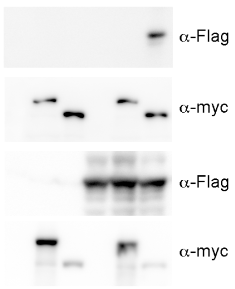

Fig. 3a

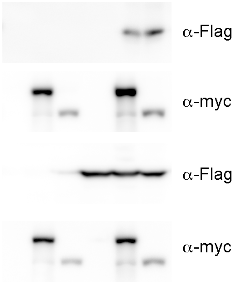

Fig. 3b

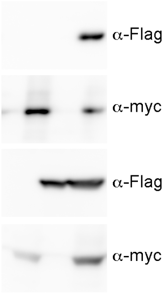

Fig. 3c

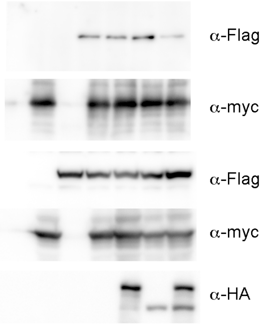

Fig. 3d

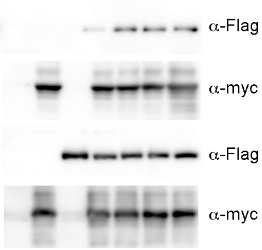

Fig. 3e

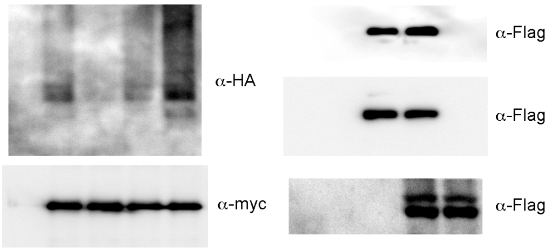

Fig. 3f

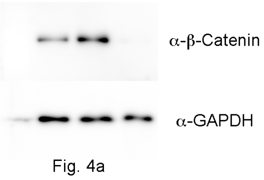

Fig. 4a

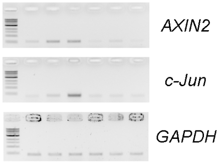

Fig. 4b

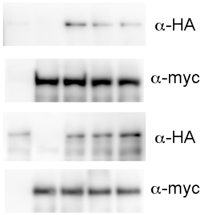

Fig. 4c

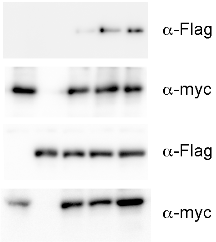

Fig. 4d

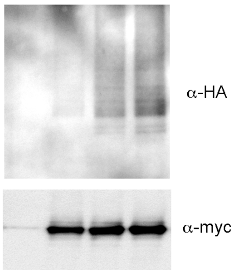

Fig. 4e

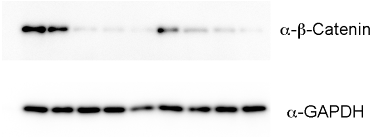

Fig. 4f

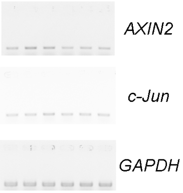

Fig. 4g

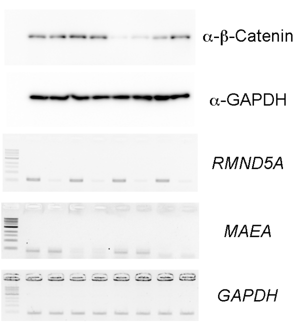

Fig. 4h

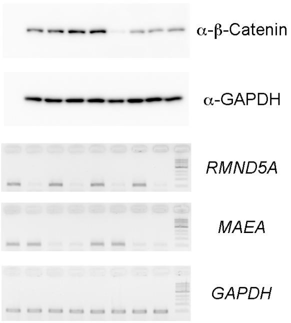

Fig. 4i

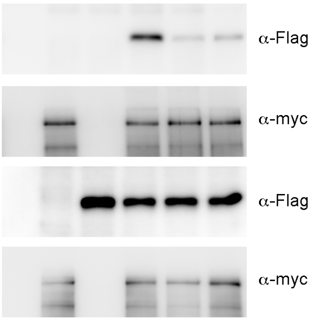

Fig. 4j

Supplementary Figure 6 continued

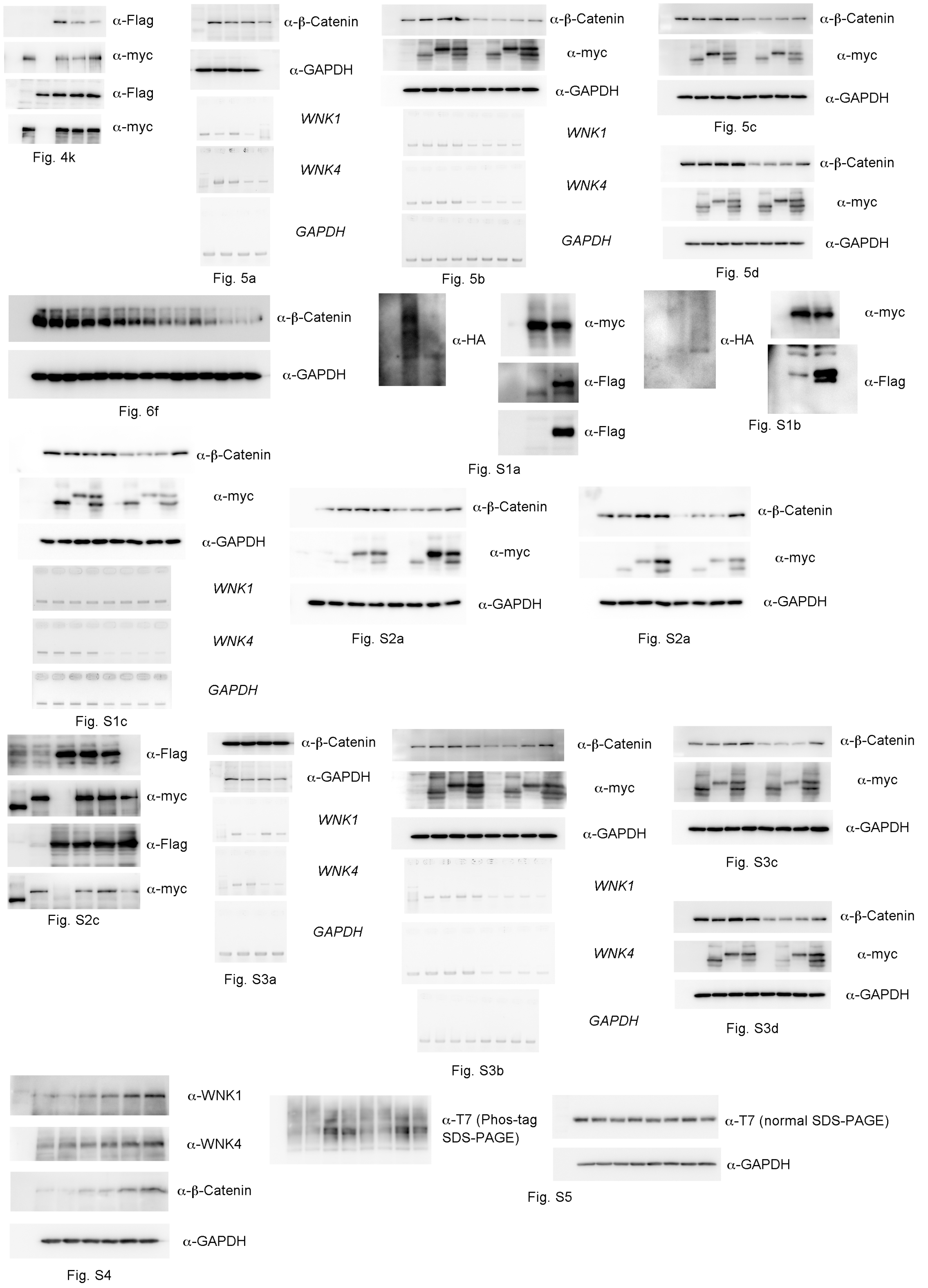

Supplementary Figure 6. Uncropped immunoblot and gel images. Uncropped

original images in all Figures and Supplementary Figures

Supplementary Table 1

Gene name	Target sequence
Human WNK1	GCAGGAGTGTCTAGTTATA
Human WNK4	GAAATTGAAGATTTGTACA
Human Dvl1	CCTACAAATTCTTCTTTAA
Human Dvl2	CCACTTTCTCCTACCAATA
Human Dvl3	GCTATAAGTTCTTCTTCAA
Human β-Catenin	GTTATCAGAGGACTAAATA
Human βTrCP	GTATTTATTCAAAAACAAA
Human FBXW11	TGATAATGACACATTCGTG
Human SIAH1	GTACTTTTCCCCTGTAAAT
Human SHPRH	TATATGGATTATAGTAGAG
Human RMND5A	CCATTTGTGGAGTTAAATA
Human MAEA	ACGACTTTATCATCTTGAC

Supplementary Table 1. Target sequence of siRNA. 19bp target sequence of siRNA we used. We ordered 21 base RNA with 2 nucleotides 3' overhang sequence for both guide and passenger strands.

Supplementary Table 2

Gene name	Sequences	
GAPDH	GCCATCACTGCCACCCAGAAGACTG	CATGAGGTCCACCACCCTGTTGCTG
Human WNK1	AAGTTAGAGCTGCGACGACTACGAG	GGTGCAGAGAACTTCCTTGCCATTC
Human WNK4	CCAAGTGACTTCATCCAAGGAACCG	TCAGAGAGTTCCTTCGCATGATGCC
Human Axin2	ACAACAGCATTGTCTCCAAGCAGC	GTCATGGACATGGAATCATCCGTC
Human c-Jun	AACCTCAGCAACTTCAACCC	ACCTGTTCCCTGAGCATGTT
Human DVL1	CAGAGCACCTCATCCAGACTCATCC	GCTCATGTCACTCTTCACCGTCAGC
Human DVL2	AACCGAGTCAGTAGTGTCACTGAGG	CAGCGTCATCGTTGCTCATGTTCTC
Human DVL3	GGACTCCACCATGTCACTCAACATC	ATGGACAAGTGGAAGTCGTCTAGGC
Human β-Catenin	AAGACATCACTGAGCCTGCCATCTG	TGGCTCCCTCAGCTTCAATAGCTTC
Human βTrCP	AGCGAATTCTCACAGGCCATACAGG	GTCCCTGTACTGCAAACAGGCAATG
Human FBXW11	GCAGCGAGTGATCTCAGAAGGAATG	GAACAGGTCACCATCAGTCCATTGC
Human SIAH1	CGCAACTTGGCTATGGAGAAAGTGG	CAGCTTGCTTGCGTGTTCCTATCAG
Human SHPRH	ATGGCTCTGAGGAATCGTGTGTCTG	TCCTCCTCCTGGTTTGCTTTCTCTG
Human RMND5A	AGACATCCACAGCAGTGTTTCTCGG	CACAGATATCAGCCCACTGGTTTGC
Human MAEA	TCGAGCACCTCAAAGAGCATAGCAG	GTTGTCGTACCGGAACTGCTGGATC

Supplementary Table 2. Sequences of primers. All primers are located in coding sequence.
