# Supplementary material for: WNK regulates Wnt signalling and β-Catenin levels by interfering with the interaction between β-Catenin and GID
Source: Commun Biol. 2020 Nov 12;3:666. doi: 10.1038/s42003-020-01386-2 (PMC7665214; doi:10.1038/s42003-020-01386-2)
Supplement: Supplementary file 3 — Description of Additional Supplementary Files [file 42003_2020_1386_MOESM3_ESM.docx]

**Description of Additional Supplementary Files**

Supplementary Data 1: Source data for all graphs.
