# Supplementary material for: WNK regulates Wnt signalling and β-Catenin levels by interfering with the interaction between β-Catenin and GID
Source: Commun Biol. 2020 Nov 12;3:666. doi: 10.1038/s42003-020-01386-2 (PMC7665214; doi:10.1038/s42003-020-01386-2)
Supplement: Supplementary file 5 — Reporting Summary [file 42003_2020_1386_MOESM5_ESM.pdf]

nature research

Corresponding author(s): Hiroshi Shibuya

Last updated by author(s): Oct 11, 2020

Reporting Summary

Nature Research wishes to improve the reproducibility of the work that we publish. This form provides structure for consistency and transparency in reporting. For further information on Nature Research policies, see our Editorial Policies and the Editorial Policy Checklist.

Statistics

For all statistical analyses, confirm that the following items are present in the figure legend, table legend, main text, or Methods section.

n/a	Confirmed	
[ ]	[x]	The exact sample size (*n*) for each experimental group/condition, given as a discrete number and unit of measurement
[ ]	[x]	A statement on whether measurements were taken from distinct samples or whether the same sample was measured repeatedly
[x]	[ ]	The statistical test(s) used AND whether they are one- or two-sided<br>*Only common tests should be described solely by name; describe more complex techniques in the Methods section.*
[x]	[ ]	A description of all covariates tested
[x]	[ ]	A description of any assumptions or corrections, such as tests of normality and adjustment for multiple comparisons
[x]	[ ]	A full description of the statistical parameters including central tendency (e.g. means) or other basic estimates (e.g. regression coefficient) AND variation (e.g. standard deviation) or associated estimates of uncertainty (e.g. confidence intervals)
[x]	[ ]	For null hypothesis testing, the test statistic (e.g. *F*, *t*, *r*) with confidence intervals, effect sizes, degrees of freedom and *P* value noted<br>*Give P values as exact values whenever suitable.*
[x]	[ ]	For Bayesian analysis, information on the choice of priors and Markov chain Monte Carlo settings
[x]	[ ]	For hierarchical and complex designs, identification of the appropriate level for tests and full reporting of outcomes
[x]	[ ]	Estimates of effect sizes (e.g. Cohen's *d*, Pearson's *r*), indicating how they were calculated

Our web collection on statistics for biologists contains articles on many of the points above.

Software and code

Policy information about availability of computer code

Data collection	Applies Biosystems 7300 Real-Time PCR Cycler (ABI) for quantitative PCR, LAS-4000 mini (GE) for image analyze and Vi-CELL (Beckman) for cell counts are used.
Data analysis	Microsoft Excel (Microsoft) and StatPlus (AnalystSoft) are used for statistc analysis.

For manuscripts utilizing custom algorithms or software that are central to the research but not yet described in published literature, software must be made available to editors and reviewers. We strongly encourage code deposition in a community repository (e.g. GitHub). See the Nature Research guidelines for submitting code & software for further information.

Data

Policy information about availability of data

All manuscripts must include a data availability statement. This statement should provide the following information, where applicable:

- Accession codes, unique identifiers, or web links for publicly available datasets
- A list of figures that have associated raw data
- A description of any restrictions on data availability

The material used in this study as #13 is available from Hiroyuki Kagechika upon reasonable request. The other data are available from the corresponding author upon request. Source data behind the graphs are available in Supplementary Data 1. All full immunoblot and gel images are shown in Supplementary Figure 6.

Field-specific reporting

Please select the one below that is the best fit for your research. If you are not sure, read the appropriate sections before making your selection.

- [x] Life sciences
- [ ] Behavioural & social sciences
- [ ] Ecological, evolutionary & environmental sciences

For a reference copy of the document with all sections, see nature.com/documents/nr-reporting-summary-flat.pdf

Life sciences study design

All studies must disclose on these points even when the disclosure is negative.

Sample size	All sample size was larger than three in each experiments. No method was used to determine sample size.
Data exclusions	No data were exclusions.
Replication	All experiments included independent biological replicates.
Randomization	All the mice used in experiments were assigned randomly.
Blinding	Investigator were not blinded during experiment.

Reporting for specific materials, systems and methods

We require information from authors about some types of materials, experimental systems and methods used in many studies. Here, indicate whether each material, system or method listed is relevant to your study. If you are not sure if a list item applies to your research, read the appropriate section before selecting a response.

Materials & experimental systems

n/a	Involved in the study	
[ ]	[x]	Antibodies
[ ]	[x]	Eukaryotic cell lines
[x]	[ ]	Palaeontology and archaeology
[ ]	[x]	Animals and other organisms
[x]	[ ]	Human research participants
[x]	[ ]	Clinical data
[x]	[ ]	Dual use research of concern

Methods

n/a	Involved in the study	
[x]	[ ]	ChIP-seq
[x]	[ ]	Flow cytometry
[x]	[ ]	MRI-based neuroimaging

Antibodies

Antibodies used	Anti-β-Catenin (ab32572, Abcam); Anti-Flag (PM-020, MBL); Anti-HA (561, MBL); Anti-myc (562, MBL); Anti-WNK1 (4979S, CST); Anti-WNK4 (5713S, CST); Anti-myc (2276S, CST); Anti-GAPDH HRP conjugated (016-25523, Wako); Anti-Rabbit HRP conjugated (NA934, Cytiva).
Validation	Anti-β-Catenin (ab32572, Abcam) : WB https://www.abcam.co.jp/beta-catenin-antibody-e247-chip-grade-ab32572.html<br>Anti-Flag (PM-020, MBL) : WB https://ruo.mbl.co.jp/bio/dtl/A/?pcd=PM020<br>Anti-HA (561, MBL) : WB and IP https://ruo.mbl.co.jp/bio/dtl/A/?pcd=561<br>Anti-myc (562, MBL) : WB https://ruo.mbl.co.jp/bio/dtl/A/?pcd=562<br>Anti-WNK1 (4979S, CST) : WB https://www.cellsignal.jp/products/primary-antibodies/wnk1-antibody/4979<br>Anti-WNK4 (5713S, CST) : WB https://www.cellsignal.jp/products/primary-antibodies/wnk4-antibody/5713<br>Anti-myc (2276S, CST) : IP https://www.cellsignal.jp/products/primary-antibodies/myc-tag-9b11-mouse-mab/2276<br>Anti-GAPDH HRP conjugated (016-25523, Wako) : WB https://labchem-wako.fujifilm.com/jp/product/detail/W01W0101-2552.html<br>Anti-Rabbit HRP conjugated (NA934, Cytiva) : WB https://www.cytivalifesciences.co.jp/catalog/0428.html

Eukaryotic cell lines

Policy information about cell lines

Cell line source(s)	Human embryo kidney (HEK293T)<br>Human colon cancer (SW480 cells, HCT116, and DLD1 cells).
Authentication	All cells were from ATCC.

Mycoplasma contamination	All cells were tested ATCC.
Commonly misidentified lines (See ICLAC register)	No cell line were defined as misidentified cell line.

Animals and other organisms

Policy information about studies involving animals; ARRIVE guidelines recommended for reporting animal research

Laboratory animals	Five-week-old BALB/cAJ1-nu/nu male mice were used for Xenograft model
Wild animals	No wild animals was used in this study.
Field-collected samples	This study does not involve samples collected from field.
Ethics oversight	Protocols using this study was approved by Ethics Committee on Animal Experiments of Nippon Medical School.

Note that full information on the approval of the study protocol must also be provided in the manuscript.
